# Contributions of cortical feedback to sensory processing in primary visual cortex

**DOI:** 10.3389/fpsyg.2014.01223

**Published:** 2014-11-06

**Authors:** Lucy S. Petro, Luca Vizioli, Lars Muckli

**Affiliations:** Centre for Cognitive Neuroimaging, Institute of Neuroscience and Psychology, University of GlasgowGlasgow, UK

**Keywords:** V1, feedback, fMRI, vision, electrophsyiology

## Abstract

Closing the structure-function divide is more challenging in the brain than in any other organ (Lichtman and Denk, 2011). For example, in early visual cortex, feedback projections to V1 can be quantified (e.g., Budd, 1998) but the understanding of feedback function is comparatively rudimentary (Muckli and Petro, 2013). Focusing on the function of feedback, we discuss how textbook descriptions mask the complexity of V1 responses, and how feedback and local activity reflects not only sensory processing but internal brain states.

## IS V1 (SOMETIMES) AT THE TOP OF THE HIERARCHY?

The era of Mountcastle, Hubel and Wiesel had “profound physiological implications” for the study of cortical processing (see Kandel, 2014). Hubel and Wiesel (1959) characterized the response properties of visual cortical neurons in columns: V1 neurons respond to their selective stimulus (e.g., a line of a certain orientation), and are embedded in a cortical architecture that exposes a functional map of columnar orientation preference and ocular dominance. These milestone findings furnished the (still current) textbook accounts of V1, which are dedicated to the feedforward cascade of processing and biased to neuronal spiking as recorded in electrophysiology. However, owing to increasingly sophisticated methodologies to assess functional responses, such as high-resolution magnetic resonance imaging or optogenetics combined with electrophysiology, this feedforward model of V1 can be updated to incorporate the rich response properties conferred by cortical feedback.

Neurons in early visual areas do not act as linear feature detectors when faced with complex inputs such as natural scenes, emphasizing the contribution of response modulation beyond the classical receptive field (Kayser et al., 2004). For example, non-linear receptive field models using natural stimuli predict V1 activity more optimally than a model fit using grating stimuli (David et al., 2004); V1 responses to bars embedded in a natural scene are reduced compared to bars on a uniform background (MacEvoy et al., 2008); and during natural scene viewing, the surround, local field potential (LFP) and spike history contribute to V1 spiking almost as much as the classical receptive field (Haslinger et al., 2012). Furthermore, V1 neurons are active even during occlusion (Sugita, 1999; Lee and Nguyen, 2001), revealing that non-stimulus-driven inputs allow early neurons to respond even to stimuli which are inferred but not directly presented to the retina. Early visual neurons therefore do not only transform retinal signals, but integrate top–down and lateral inputs, which convey prediction, memory, attention, reward, task, expectation, locomotion, learning, and behavioral context. Such higher processing is fed back (monosynaptically or otherwise) to V1 from cortical and subcortical sources (Muckli and Petro, 2013). Understanding the function of feedback has implications not only for vision, but for structural and dynamic networks for cognition and behavior (Harris and Mrsic-Flogel, 2013). Indeed, Gilbert and Li (2013) suggest that each cortical neuron is a “microcosm of the brain as a whole, with synapses carrying information originating from far flung brain regions.” Top–down influences modulate feedforward (classical) receptive fields and also many of the contextual interactions performed by intrinsic V1 neurons. Here, we discuss some effects of top–down inputs to V1, culminating in the tempting speculation that V1 is misplaced as merely the earliest, sensory stage of the visual cortical hierarchy.

## NON-GENICULATE INPUT TO V1 – INTERNAL PROCESSING

Aptly, the visual brain is classically studied by presenting it with visual stimuli, revealing extrinsically driven receptive fields in V1. However: (1) sensory areas are neither monomodal (e.g. Vetter et al., 2014) nor immune to higher processes; (2) feedback and lateral inputs outnumber feedforward inputs and (3) the brain is now more commonly referred to as a parallel rather than serial processor (Singer, 2013). Much can therefore be learned about intrinsically driven “response fields” in V1 (Muckli, 2010), and there is abundant evidence that V1 is involved in processing distinct from the classical feedforward activation that defines its position as the first cortical stage of vision. The reciprocal nature of the visual system suggests that in fact, in an inversion of sensory processing, visual scenes can be back-projected to V1 (Harth et al., 1987). If so, intuitively this “internal vision” would be accessible in V1 during sleep or mental processing, i.e., when there is no feedforward input. It is possible to study internal processing by examining V1 in the absence of feedforward activation, such as in visual occlusion (Smith and Muckli, 2010) or illusion (Lee and Nguyen, 2001; Muckli et al., 2005; Murray et al., 2006; Weigelt et al., 2007; Maus et al., 2010; Kok and de Lange, 2014), in the blind (e.g., Amedi et al., 2004), blindfolded (Vetter et al., 2014) or sleeping (Horikawa et al., 2013), and during working memory, (Harrison and Tong, 2009), imagery (Albers et al., 2013) and expectation (Kok et al., 2014). During eyes-closed, resting state functional magnetic resonance imaging (fMRI), hyperactive V1 has been observed in individuals with posttraumatic stress disorder who score highly on scales for re-experiencing (Zhu et al., 2014). In addition to feedback from higher visual areas, such as during occlusion or illusion, top–down influences signal behavioral context so that V1 neurons respond adaptively to the functional state of the brain (Gilbert and Li, 2013). We discuss higher processing that can be read out in V1, and suggest that not only is V1 activity linked to higher vision, but to brain states such as attention or expectation (that are determined by network interactions, Park and Friston, 2013) and tasks (Petro et al., 2013).

### PREDICTION

A great deal is established about which external inputs make visual neurons spike. In contrast, less is known about the inputs which do not directly signal feedforward information transmission. One eminent theory is that feedback is actively involved in the analysis of feedforward signaling. Feedback may perform hypothesis-testing by transmitting Bayesian priors generated from memory or internal models down the visual hierarchy (e.g. Lee and Mumford, 2003). For example, one candidate mechanism for perceptual inference is that of predictive coding, in which descending predictions arising from deep pyramidal cells are compared to incoming sensory signals, and the computed mismatch (prediction error) is transferred in the feedforward stream of the superficial pyramidal cells up to the next higher cortical level to update internal models (reviewed in detail Friston, 2005; Clark, 2013). Several models in which neurons engage in probabilistic processing in order to infer the causes of their inputs have been proposed (e.g., Rao and Ballard, 1999; George and Hawkins, 2009; Lochmann and Deneve, 2011; Dura-Bernal et al., 2012), posing a challenge to feedforward theories of vision. The role of internal models in mediating predictive processing has been suggested by data from ferret V1, where, over development, spontaneous activity becomes increasingly similar to the activation induced by natural scenes (Berkes et al., 2011). This indicates that intrinsic (spontaneous) activity is akin to the responses that were previously experienced. Furthermore, when the visual flow of grating stimuli is selectively de-coupled to the rate at which a mouse runs on a ball, neurons in layer II/III of V1 signal the mismatch between actual visual flow feedback and that predicted by locomotion (Keller et al., 2012), which could be the putative error signal in V1. Visual evoked potentials in mouse V1 have been shown to be specific to previously learned spatiotemporal sequences of grating stimuli, and are even predictive of individual sequence elements during omissions (Gavornik and Bear, 2014). Furthermore, there are experimental observations indicating cortical prediction in human V1. Using fMRI, Alink et al. (2010), measured a reduction in blood oxygen level dependent (BOLD) signal to spatiotemporally predictable stimulation. This reduction is consistent with the suppression of predictable inputs in lower levels by feedback from higher areas (in this instance, V5; Vetter et al., 2013). Such observations are tailored to the assumptions made by predictive coding, and it is known that the hemodynamic signal is sensitive to top–down afferents to V1 (Logothetis, 2008; Muckli, 2010). However, relating theoretical models with empirical data will require more invasive strategies. Techniques such as optogenetic fMRI (ofMRI, which permits the study of neuronal function whilst measuring brain activity, Lee et al., 2010) promise to shed light on how to extrapolate from the macroscopic level of the BOLD signal to the microscopic level of neurons prescribed in the predictive coding framework, during testable visual stimulation.

Theories of cortical prediction are elegant, biologically conceivable and mathematically valid, however, they remain data-modest in early visual cortex. We identify at least two key areas that require substantiation: (1) How are predictions and errors implemented by V1 neurons? Models of prediction are constrained by anatomy (cortical laminae, feedback/feedforward projections, cell subtype, e.g. local GABAergic inhibitory interneurons and long-range glutamatergic excitatory neurons, and synaptic physiology), but it remains theoretical to what extent or how V1 neurons implement prediction in their ion channels, membrane voltage, and synapses (see Fiorillo, 2008). Furthermore, (2) how does the abstract language spoken by higher areas translate to the detailed language of V1 neurons? V1 projects upwards a fine-grained representation, which becomes increasingly invariant as it advances the hierarchy, but it is unclear how abstract representations are transmitted back down the hierarchy. If feedback contains probabilities or predictions of sensory inputs, and V1 assimilates these with the actual sensory inputs, then V1 is best conceptualized as an interactive hierarchical loop and not as a “first pass analysis” (Lee et al., 1998). How sensory inputs, which signal detail, are combined with internal templates, which may signal predicted means or variances of sensory details, needs to be tested further. A candidate for the integration of feedforward and feedback signals is back-propagation-activated calcium signaling (BAC; Larkum, 2013). The anatomical substrate of this “BAC” mechanism is the layer I tuft dendrites of the pyramidal cells which reside in layer V. Vast feedback inputs arrive to these tuft dendrites, triggering Ca^2+^ spikes proximal to the apical dendrites. The consequence of these dendritic Ca^2+^ spikes is that feedback inputs may dictate the firing of the pyramidal neuron far more than was previously thought. Via this Ca^2+^ spiking mechanism, the response to feedforward somatic input (or sensory signals) is strengthened if it matches the contextual inputs or internal predictions to the tuft dendrites, e.g., it can convert a single somatic output spike into a 10 ms burst containing 2–4 spikes. The discovery of this associative mechanism illuminates one “crowning mystery” of cortex, that is, layer I (Hubel, 1982).

Observations of BAC firing impose constraints on models of how pyramidal neurons accomplish predictive coding. During BAC signaling, the predictable information is *amplified*. However, under rules of predictive coding, feedback acts to *suppress* activity in the preceding area of cortex. The common detail between predictive coding and BAC signalling is apparent in the laminar organization of predictive coding: deep layer 5 pyramidal neurons are the “prediction units,” the same as is described in mechanisms of BAC signaling. However, BAC signaling suggests that predictable inputs are amplified within a single neuron, whereas predictive coding may engage computations within columnar circuitry for an overall effect to silence predictable inputs to an area. Therefore, although predictive coding and BAC overlap insofar as deep pyramidal neurons signal predictions, it remains to be seen how these amplified predictions within a layer 5 neuron contribute within a column (or area) to suppress prediction error in layer 2/3 (the “prediction error units” in predictive coding) before residual errors are sent up the hierarchy. Preparations which can measure dendritic signaling will contribute to resolving this question, and more generally are an exciting prospect for future explorations of V1 neurons which receive only feedback inputs, i.e., during occlusion or expectation (prior to stimulation).

### MEMORY

Given the fine-grained and retinotopic nature of V1, it is a candidate region for the maintenance of high-resolution information during working memory or reactivation during episodic memory. Spatially specific working memory representations in V1 have been demonstrated by the successful decoding of grating stimuli during a retention period in the cortical location of their original representation (Pratte and Tong, 2014). The information maintained in working memory that is represented in V1 reflects the relevance of items, and this can be causally interrupted using transcranial magnetic stimulation (TMS) (Zokaei et al., 2014). In a memory-color paradigm, successful cross-classification of V1 activity patterns between colored hues and gray scale objects associated with those hues, was interpreted as the result of the feedback of prior knowledge to V1 (Bannert and Bartels, 2013). The capacity of visual memory for object details is great (Brady et al., 2008); these details may be stored as early as V1 and reactivated by feedback, contingent on behavioral demands. The reactivation of V1 may be related to top–down influences from the hippocampus for successful memory consolidation during sleep. Firing sequences evoked during awake experience are replayed in both V1 and the hippocampus during sleep phases in the mouse (Ji and Wilson, 2007). Furthermore, human hippocampus activity covaries with early visual activity, which predicts the information that subjects retrieve from memory (Bosch et al., 2014). The hippocampus exerts top–down effects on early vision during scene extrapolation (Chadwick et al., 2013), prompting new theories of hippocampal memory whereby it constructs the “world beyond the immediate sensorium” (Maguire and Mullally, 2013). The recruitment of V1 by the hippocampus to construct the world would appear functional, given that V1 depicts the visual environment with the highest resolution.

### REWARD

V1 was not classically thought to play an essential role in reward processing. However, a number of studies indicate that reward modulates the representation of features in V1. V1 neurons in the rat have been shown to signal value (Shuler and Bear, 2006), and more recent calcium-imaging data from mouse V1 reveals that the association between stimulus and reward alters response amplitude in stimulus-specific assemblies (Goltstein et al., 2013). Neurons in macaque V1 that signal value also exhibit strong attentional effects (Stǎnişor et al., 2013) and future studies will clarify the role of feedback in this overlap. Cholinergic input to V1 from the basal forebrain of the rat modulates specifically the learning of reward timing, but not the expression of previously learned cue-reward intervals (Chubykin et al., 2013). In human early visual cortex, value is encoded across populations of neurons, in which response profiles are sharpened (Serences and Saproo, 2010). Anticipatory activity in V1 may in some instances be driven by dopaminergic input directly from the ventral tegmental area (Phillipson et al., 1987; Tan, 2009) or indirectly from the prefrontal cortex (Noudoost and Moore, 2011). Anticipatory haemodynamic signals in V1 are found even without feedforward stimulation (Sirotin and Das, 2009). Such baseline shifts point to the “dark matter” of the brain, that is, much can be learned from the substantial energy consumption of neurons even during resting states (Shoham et al., 2006; Raichle, 2011). In an elegant design to exclude the effects of anticipation (as well as attention and expectation), it was shown that the effects of dopaminergic reward on V1 can decrease its activity (Arsenault et al., 2013). Further experiments will elucidate if this decrease equates to a sharpened representation of rewarding stimuli, and more generally, the role of cholinergic, dopaminergic, and feedback mechanisms in reward effects in V1.

### VISION FOR ACTION AND VISUAL PERCEPTION

Feedback to V1 has a role in how we perceive and interact with the visual world. For example, reciprocal feedback from parietal portions of the dorsal stream to early visual areas is likely involved in visuospatial processing, although the function of these networks remains to be fully elucidated. The dorsal stream is activated during reaching and grasping, and Ban et al. (2013) offer the thought-provoking idea that early visual cortex interacts with other sensory modalities (e.g. tactile or motor), as an implicit representation of an occluded object in visual cortex could facilitate the touching or grasping of the occluded portion of the object. In addition, top–down input, likely from auditory cortex or association areas, leads to categorical activation in early visual cortex (Vetter et al., 2014) during natural sound processing in blindfolded subjects. Such activity in visual cortex could be biased by higher areas to the feature content or localisation of content in a visual scene, or, with motor guidance, aid in visually orienting to the source of auditory signals. Motor inputs related to locomotion are sufficient to drive V1 activity; Keller et al. (2012) observed motor-related activity in mouse V1, without any visual input. V1 neurons responded when the mouse was running on a ball during complete darkness, and this activity was comparable to that evoked by visual stimulation with gratings. Further studies will clarify the involvement of cortical feedback in visuo-motor processing and the sensory guidance of movement, and the recruitment of V1 in these processes.

The ventral visual stream is concerned with detailed form representation, and the importance of feedback in the ventral stream or “recurrent occipitotemporal network” (Kravitz et al., 2013) is linked to the retinotopic organization of V1. For example, objects presented in the periphery trigger feedback to foveal V1 where object detail is processed (Williams et al., 2008), and interrupting this feedback using TMS at a relatively late time interval impairs peripheral object perception (Chambers et al., 2013). In contrast, feedback related to scene processing is back-projected to the periphery of V1 (Smith and Muckli, 2010). A causal role of recurrent processing in the ventral stream suggests that late activation in V1 contributes to scene categorization (Koivisto et al., 2011). During face processing, feedback (putatively from temporal cortex) task-dependently biases retinotopic sub-regions of V1 responding to certain features (Petro et al., 2013). Perceptual expectation can enhance the representation of stimuli in V1 whilst at the same time suppressing V1 (Kok et al., 2012), in line with predictive coding theories of dampening predicted inputs. Within both dorsal and ventral streams, recurrent feedback loops might be critical for conscious processing (Lamme and Roelfsema, 2000; Dehaene and Changeux, 2011).

Effects of feedback to V1 on visual perception have also been studied more invasively, contributing to the mechanistic understanding of feedback. Enhanced visual discrimination is seen in awake behaving mice after optogenetically activating cholinergic neurons projecting to V1 from the basal forebrain (Pinto et al., 2013), with a probable role in attentional function. During scene processing, population codes in mouse V1 become increasingly sparse compared to viewing control scenes lacking statistical regularities (Froudarakis et al., 2014). This encoding by a smaller set of neurons only when the scenes were not phase-scrambled fits with theories of back-projected predictions suppressing feedforward processing, or could be related to microcircuits within V1. The study of Berkes et al. (2011) mentioned previously hints that the cortex utilizes a strategy of decreased processing for experienced or expected signals. In ferret V1, it was found that across development spontaneous activity begins to reflect the activity evoked by natural scenes, and therefore prior expectations. This increasing similarity between evoked and spontaneous activity reveals that the cortex updates its internal model with experience, with predictive coding theories suggesting that these internal models are used to generate predictions of sensory input, which can supress activity at early cortical levels. Intra-areal, inhibitory interneurons can also modulate visual perception. By optogenetically targeting parvalbumin-positive interneurons in V1, Lee et al. (2012) revealed that these neurons are involved in sharpening feature selectivity and in perceptual orientation discrimination. Parvalbumin neurons are targeted by feedback, and top–down connections putatively control inhibitory activity as dictated by behavioral demands (possibly by inhibiting predictable inputs). In neuropathophsyiology, *N*-methyl-D-aspartate (NMDA) receptor hypofunction on parvalbumin neurons interferes with gamma oscillations, which is linked to schizophrenia and depression (Gonzalez-Burgos and Lewis, 2012; Phillips and Silverstein, 2013). Attenuated visual illusion effects observed in schizophrenia might relate to an interruption of top–down predictions (see Notredame et al., 2014). These predictions are maintained in healthy populations who experience the illusions.

Of the aforementioned studies, the data on visual perception are conceivably related to predictions from higher cortical visual areas. This predictive processing may be temporally discernible from that of attention, and have sources in independent regions from those that allocate attention. In contrast, it is less intuitive to associate feedback with prediction during vision for action, without knowing more about the cortico-cortical connections crossing domains from motor to visual. However, prediction is assumed to be a general function of the cortex and motor actions are often highly repetitive and structured (and therefore predictable). Anatomical connections reveal that it is essential to include the contribution of subcortical pathways and the cerebellum in predictive feedback during sensorimotor processing. For example, the cerebellum is involved in generating predictions of the sensory consequences of actions (Kawato and Wolpert, 1998), which may also be represented in V1. The cerebellum is also involved in predictions of perception (Roth et al., 2013), suggesting that, like cortex, the cerebellum’s role in prediction is unspecific to any one processing domain. It remains unknown how V1 and the cerebellum interact during perception, and what role feedback has in this processing.

## A NEW LANDSCAPE OF V1

For several years, neuroscience has yielded abundant data on the intricate workings of V1. Yet, this unique cortical area remains, in many ways, a mystery. The gain of modern experimentation is that, with advancing imaging and recording techniques, we can understand the (cellular) mechanics of V1. The reward for venturing “under the hood,” will be to learn if theoretical concepts of cortical feedback can be realized in corresponding biological substrates. Hence, decades after the revolutionary work of Hubel and Wiesel (1959), there are continued efforts to understand V1. For example, it was found that in the macaque, the majority of feedback to V1 arises from V2, where axons arborize in supragranular layers I and II, and infragranular layer V (Rockland and Virga, 1989), and more recently it has been shown that these axons fed back from V2 to V1 differ in their bouton morphology (axons forming bouton clusters or studded continuously with boutons in layer I, or forming en passant boutons in layers III and V) and postsynaptic density size (Anderson and Martin, 2009). Rodent models of V1 lend themselves to innovative invasive approaches, allowing countercurrent visual processing streams to be studied on the cellular level. For example, using subcellular Channelrhodopsin-2-assisted circuit mapping and patch clamp recordings, Yang et al. (2013) showed that depolarizing feedback input is balanced between parvalbumin interneurons and pyramidal neurons in layer II/III of mouse V1. This balance is in contrast to feedforward pathways (which provide substantially more depolarizing input to layer II/III parvalbumin neurons than to excitatory pyramidal cells) and therefore has implications for pathway-specific excitation/inhibition. In mouse layer V, feedback input to tuft dendrites leads to NMDA spikes which (by supporting calcium spikes, which are “tremendously explosive”) are thought to be critical for the integration of top–down inputs in cortex (Larkum et al., 2009; Larkum, 2013). Furthermore, it becomes increasingly clear that we must “reach beyond the classical receptive field” (Angelucci and Bullier, 2003) because feedback inputs bestow the full range of center and surround receptive field properties to V1 neurons (in combination with feedforward and lateral inputs; Angelucci and Bressloff, 2006). For example, in the macaque, surround suppression in V1 is reduced when feedback is eliminated (Nassi et al., 2013), feedback augments V1 responses to collinear contours in the owl monkey (Shmuel et al., 2005), and during pattern motion processing, feedback modulates subthreshold influences beyond the classical receptive field, facilitating global constructs from local features represented in V1 (Schmidt et al., 2011). Moreover, the spatiotemporal receptive fields in layer II/III of macaque V1 may be best characterized by their intracortical inputs and not by their visual inputs (Yeh et al., 2009).

Markov et al. (2014) suggest that actually we have only a rudimentary understanding of connectional rules of feedback and feedforward projections. Indeed, there are thought to be two systems of feedback and feedforward projections: supragranular and infragranular (Markov and Kennedy, 2013), and future investigations will enlighten how these pathways constrain models of cortical information processing in more detail (e.g., Bastos et al., 2012). Studies of neuronal synchrony suggest gamma-band phase coherence is restricted to supragranular and beta-band to infragranular layers (see Buffalo et al., 2011; Xing et al., 2012). Top–down input in the beta or alpha band to deep layers modulates gamma activity (associated with bottom–up processing) in more superficial layers (see Spaak et al., 2012; Bastos et al., 2014, bioRxiv). In monkey V1, distinct multiunit profiles in layers corresponding to feedforward and feedback processing can be seen during the perception of figure-ground segregation (Self et al., 2013). Laminar analysis of V1 in humans, using fMRI with multivoxel pattern analysis (MVPA), shows that contextual feedback arrives to the superficial layers of cortex (Muckli, OHBM conference abstract, 2014). MVPA scrutinizes information in the multivariate pattern of activity across an array of voxels, to discriminate between stimuli or states that are potentially neglected by conventional analysis involving spatial averaging (Kriegeskorte and Bandettini, 2008). The spectrally symmetric encoding models (Gourtzelidis et al., 2005; Thirion et al., 2006; Dumoulin and Wandell, 2008; Jerde et al., 2008; Kay et al., 2008; Mitchell et al., 2008; Naselaris et al., 2009; Schönwiesner and Zatorre, 2009) can explicitly quantify the information contained in individual voxels (Naselaris et al., 2011), thus providing insights about the preferred features coded by a given voxel. Aiding in our understanding of visual cortex, these techniques have thus far been optimized within the feedforward framework. With a clearer understanding of their advantages and limitations, these approaches combined with layer-resolution fMRI have the potential to unveil a wealth of information about the functional role of feedback activity in V1 (Muckli, OHBM conference abstract, 2014; Morgan et al., OHBM conference abstract, 2014). Layer-resolution fMRI can also be combined with pharmacological intervention to assess laminar differences during tasks that are dependent on top–down processing. For example, texture discrimination, which relies on recurrent processing, is impaired subsequent to ketamine administration (Meuwese et al., 2013). Ketamine blocks NMDA receptors which are implicated in feedback processing due to their higher concentration in supragranular layers (Rosier et al., 1993), their modulatory function (Collingridge and Bliss, 1987) and their contribution to figure-ground segregation (Self et al., 2012).

## CONCLUSION

V1 is one of the best studied cortical areas in terms of its robust stimulus-response relationship. This fine-grained, feedforward propagation of the visual world is V1’s principle function. However, increasing evidence reveals a more complex and comprehensive account of V1: through intrinsic and feedback connections, V1 neurons are also capable of complex visual (scene analysis) and non-visual (cognitive) responses. One function of feedback may be to flexibly “set the system” according to present behavioral requirements, i.e., distribute top–down influences even to the earliest sensory areas. We have highlighted some higher-order processes that can be read-out from V1. During active vision, feedback may transmit Bayesian inferences of forthcoming inputs to V1, to facilitate perception. Feedback may also sharpen the representation of rewarding stimuli in V1. During sleep, V1 might be involved in the higher-order replay of experienced events, for memory consolidation. With growing capabilities to study the brain at molecular, cellular, systems, behavioral and cognitive levels, one hopes that future developments will clarify the role of V1 neurons as adaptive responders, and elucidate how internal brain states regulate sensory processing in V1.

## Conflict of Interest Statement

The authors declare that the research was conducted in the absence of any commercial or financial relationships that could be construed as a potential conflict of interest.
